# Editorial: Perspectives in non-invasive brain stimulation and neuromodulation

**DOI:** 10.3389/fnhum.2023.1324517

**Published:** 2023-11-03

**Authors:** Joaquim P. Brasil-Neto

**Affiliations:** Centro Universitário Unieuro, Brasília, Brazil

**Keywords:** brain stimulation, transcranial magnetic stimulation (TMS), transcranial direct current stimulation, neuromodulation, non-invasive

This Research Topic attempts to bring together cutting-edge aspects in the field of non-invasive brain stimulation (NIBS) and neuromodulation. Its different contributions focus on various aspects of transcranial stimulation, but a recurring theme is that of a search for neurophysiological markers that could confirm the response to treatment and/or predict such responses in individual subjects (Sanches et al., 2020). Another important area of study is the role of electrode montages on the results obtained by different researchers (Evans et al., 2022). Finally, the great volume of experimental data already available in the literature on NIBS presents a challenge to any kind of literature review, and the advent of bibliometric techniques might help researchers make sense of this vast material (Zheng et al., 2020). On the other hand, such wealth of experimental data may also allow the proposal of physiologically sound hypotheses to support innovative experimental and therapeutic protocols targeting various neurological and neuropsychiatric conditions (Fregni et al., 2021).

Regarding neurophysiological markers, the combination of transcranial magnetic stimulation (TMS) and EEG may provide a useful tool to evaluate the effect of specific rehabilitation strategies in stroke patients (Chen et al., 2022), as is shown in the study by Simis et al.. Likewise, Nakamura-Palacios et al. review the literature on frontal midline theta oscillations (FM-theta) as a potential marker of NIBS effects on working memory (WM). They hypothesize that FM-theta could be a useful neurophysiological marker of neuromodulatory changes induced by NIBS in both normal volunteers undergoing working memory (WM) experiments and in patients with neuropsychiatric disorders. When using NIBS to treat patients, cortical targets need to be well-chosen. Andrade et al. have shown how resting-state EEG data, classified according to a machine learning protocol, could have a potential predictive value of tDCS treatment efficacy, when associated with cognitive treatment, in patients with Alzheimer's disease (AD). Their results suggest FC1, F8, CP5, Oz, and F7 as the most promising therapeutic targets, and their approach may serve as a guide for a patient-centered NIBS strategy in AD patients.

As to the impact of different electrode montages and of individual factors on the results of neuromodulatory techniques, Menze et al. point out that attempts to use tDCS to improve cognition have led to mixed results, and that this is likely due to the method's great susceptibility to changes in protocols as well as to individual factors. Therefore, they compared frontoparietal network stimulation (right prefrontal anode, left posterior parietal cathode) against conventional and sham tDCS in modulating working memory (WM) capacity-dependent transfer effects of a single-session distractor inhibition (DIIN) training. Their results showed that transfer effects on WM by a single-session DIIN training combined with tDCS are dependent on WM capacity. Moreover, the specific electrode montage was also shown to play a definite role in the responses.

The literature on NIBS has been growing exponentially, and refined bibliometric tools, such as scientometric mapping, may allow scientists to identify new trends in research, leading research groups, and collaboration clusters around the world, as shown by Medeiros et al.. Their study is the first attempt to provide a bibliometric perspective on the use of neuromodulation to study the motor aspects of speech.

Even with the rapid expansion of NIBS studies, however, there are still underexplored areas, and physiologically sound hypotheses are needed to guide further experiments. One of these areas is represented by cognitive impairment after severe traumatic brain injury (sTBI). tDCS appears to be useful in improving cognition and functionality in these patients (De Freitas et al., 2021), but the literature also reports negative results. Cordeiro et al. review the existing studies on this topic and, based on existing evidence on the effects of tDCS on brain processes, put forward a hypothesis that supports a real therapeutic effect of tDCS in these patients, especially in the acute and subacute phases and in combination with conventional therapy sessions.

## Author contributions

JB-N: Conceptualization, Writing – original draft, Writing – review & editing.
